# Occurrence and multilocus genotyping of *Giardia duodenalis* from post-weaned dairy calves in Sichuan province, China

**DOI:** 10.1371/journal.pone.0224627

**Published:** 2019-11-04

**Authors:** Jiaming Dan, Xueping Zhang, Zhihua Ren, Liqin Wang, Suizhong Cao, Liuhong Shen, Junliang Deng, Zhicai Zuo, Shumin Yu, Ya Wang, Xiaoping Ma, Haifeng Liu, Ziyao Zhou, Yanchun Hu, Hualin Fu, Changliang He, Yi Geng, Xiaobin Gu, Guangneng Peng, Zhijun Zhong

**Affiliations:** 1 College of Veterinary Medicine, Sichuan Agricultural University, Key Laboratory of Animal Disease and Human Health of Sichuan, Chengdu, China; 2 The Chengdu Zoo, Institute of Wild Animals, Chengdu, China; Aga Khan University - Kenya, KENYA

## Abstract

*Giardia duodenalis* is a zoonotic parasitic protist and poses a threat to human and animal health. This study investigated the occurrence of *G*. *duodenalis* infection in post-weaned calves from Sichuan province, China. Faecal samples were collected from a total of 306 post-weaned calves (3–12 months old) from 10 farms, including 4 intensive feeding farms and 6 free-ranging farms. The overall infection rate of *G*. *duodenalis* was 41.2% (126/306) based on the PCR results at any of the three genetic loci: beta-giardin (*bg*), triose-phosphate isomerase (*tpi*) and glutamate dehydrogenase (*gdh*) genes. *Giardia duodenalis* assemblages E (n = 115, 91.3%), A (n = 3, 2.4%), and A mixed with E (n = 8, 6.3%) were identified among the 126 positive specimens. Multilocus sequence typing of *G*. *duodenalis* revealed 34 assemblage E multilocus genotypes (MLGs), 1 assemblage A MLG and 7 mixed assemblage (A and E) MLGs. The eBURST data showed a high degree of genetic diversity within assemblage E MLGs. The phylogenetic tree revealed that MLG E3 was the primary MLG subtype in Sichuan province and also the most widely distributed in China.

## Introduction

*Giardia* is one of the most common parasitic protists that infects both humans and animals, poses a considerable threat to human and animal health globally [1, 2]. Among the six species of *Giardia*, only *Giardia duodenalis* can infect humans and animals (domestic, farmed and wild animals) [3, 4]. The life cycle of *Giardia* is relatively simple; involving two developmental stages of rapid multiplying trophozoites and infectious cysts, transmitted via the faecal-oral route (i.e., faeces, contaminated water or food) [1, 5]. Humans and animals infected with *Giardia* usually show symptoms such as diarrhoea, abdominal cramps, weight loss, malabsorption or recessive infections without obvious clinical symptoms [5, 6]. Young animals are more susceptible to giardiasis than adults and likely linked to the immature immune status, which lead to substantial production losses to the livestock industry [6].

*Giardia duodenalis* is recognised as a complex comprised of at least eight different assemblages (A–H) [4, 7]. Assemblage A and B can infect various mammals including humans, and are considered as the zoonotic assemblages. The other assemblages are either host specific or have narrow host ranges [6]. Cattle are dominantly infected with *G*. *duodenalis* assemblage E. Although there are fewer reports of zoonotic assemblages A and B, cattle are recognized as the contributor of the zoonotic sources of infection [6]. Many recent studies have focused on the infection of *G*. *duodenalis* in dairy calves, and the occurrence has been found to be significantly different between pre- and post-weaned stages [8–13]. In China, studies have also revealed different infection rates in pre- and post-weaned dairy calves, e.g., in Liaoning [14], Xinjiang [15], Hubei [16] and Guangdong [17] provinces. In our previous study, we conducted a preliminary study on *G*. *duodenalis* infection in pre-weaned calves in Sichuan province, China [18]. However, information regarding the occurrence in post-weaned dairy calves in Sichuan province is limited.

In this study, we further investigated the occurrence and genetic diversity of *G*. *duodenalis* in post-weaned calves from Sichuan province by using multilocus genotype (MLG) data and by assessing the zoonotic potential.

## Materials and methods

### Sample collection

**Fig 1:**
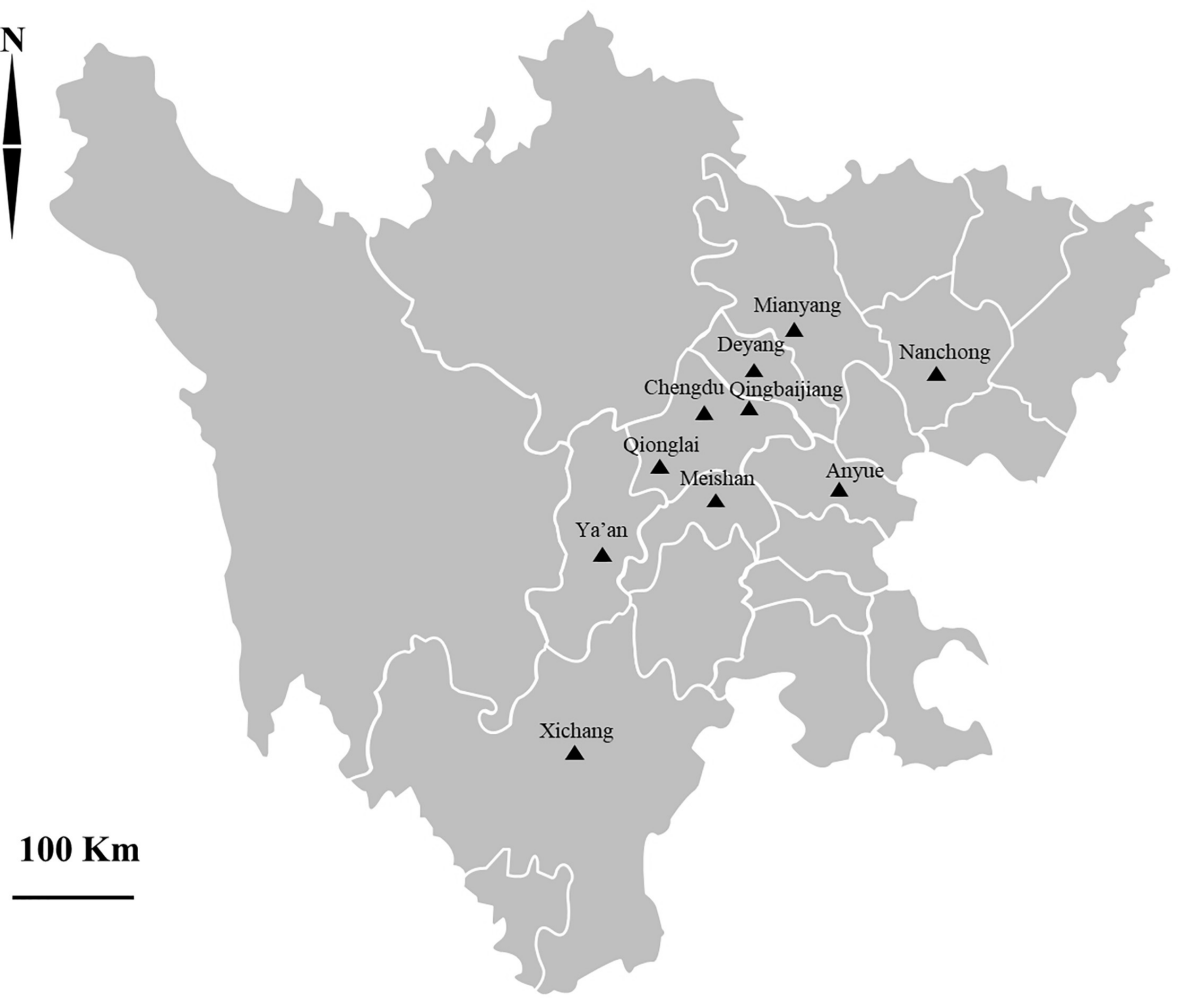
Distribution of sampling sites in Sichuan province in this study. **S**ampling sites from the present study are indicated by black triangles.

A total of 306 faecal samples were collected from post-weaned calves (3–12 months old) from 10 farms in 10 regions in Sichuan province, southwestern China, from May to November 2018 (Fig 1). At the time of faecal collections, there were no reported cases of diarrhoea in the herds but with a history of diarrhoea. The collection sites included seven of the areas from our previous study [18]: Anyue (105°33′E, 30°10′N), Chengdu (104°06′E, 30°57′N), Deyang (104°39′E, 31°13′N), Meishan (103°84′E, 30°08′N), Mianyang (104°67′E, 31°47′N), Qingbaijiang (104°25′E, 30°88′N), and Qionglai (103°46′E, 30°41′N); and three additional areas: Nanchong (106°72′E, 31°01′N), Xichang (102°51E, 28°64′N), and Ya’an (103°08′E, 30°18′N). Of the 10 farms, four (Chengdu, Mianyang, Nanchong and Qionglai) were intensive feeding farms, while the other six were free-ranging. Specific information on intensive and free-ranging farming and the specific sampling protocols used in the present study were consistent with those stipulated in our previous study [18]. A city-level map was provided by the National Geomatics Centre of China (National Geomatics Centre of China, Beijing, China, http://ngcc.sbsm.gov.cn/).

Fresh faecal samples (~25 g per calf) were collected directly from the rectum of the study calves using disposable gloves, transferred into disposable plastic bags, and then stored in 2.5% potassium dichromate at 4°C prior to DNA extraction.

This study was reviewed and approved by the Research Ethics Committee and the Animal Ethical Committee of Sichuan Agricultural University (DYY-S20174604). Permission was obtained from the farm owners before collecting the fecal samples.

### DNA extraction

Before DNA extraction, stored faeces were washed with distilled water to remove the potassium dichromate. Genomic DNA was extracted from ~250 mg of the individual samples using the PowerSoil DNA isolation kit (MOBIO, USA), according to the manufacturer’s instructions. All DNA samples were stored at −20°C prior to analysis by *Giardia* PCR.

### PCR amplification

*Giardia duodenalis* DNA was detected by nested PCR amplification of the beta-giardin (*bg*), triose-phosphate isomerase (*tpi*) and glutamate dehydrogenase (*gdh*) genes. The primers and amplification conditions used in this study have been described previously [19]. Positive and negative controls were included in each test. The secondary PCR products were visualized under UV light after electrophoresis on 1% agarose gel mixed with Golden View.

### Sequence analysis

All positive secondary PCR products were sent to BGI Tech Solutions (Liuhe Beijing) Co., Limited and were sequenced in both directions. Sequences were aligned with reference sequences from GenBank using BLAST (http://blast.ncbi.nlm.nih.gov) and Clustal X (http://www.clustal.org/).

Specimens that were successfully subtyped at all three loci were used to investigate the MLGs of *G*. *duodenalis*. Sequences were concatenated for each positive isolate to form a multilocus sequence in accordance with (*bg* + *tpi* + *gdh*). All the concatenated MLGs were used in a neighbour-joining analysis, with the Kimura-2 parameter model calculated using the Molecular Evolutionary Genetics Analysis (MEGA) version 7 (http://www.megasoftware.net/). The genetic pedigree of the assemblage E MLGs in Sichuan was assessed by using eBURST 3.0 (http://eBURST.mlst.net).

The novel *G*. *duodenalis* genotypes obtained at *bg*, *tpi* and *gdh* loci in this study were deposited in GenBank under the accession numbers: MK642904-MK642913.

### Statistical analysis

The variation in *G*. *intestinalis* prevalence among the different regions was analysed by *χ2* test using SPSS Statistics version 20.0. Differences were considered significant at *P* < 0.05.

## Results and discussion

Infected animals were detected from all of the 10 examined farms, with prevalence ranging from 6.7% to 63.3% (Table 1). This study revealed *G*. *duodenalis* as a common and widespread pathogen in post-weaned dairy calves in Sichuan province, China. Based on the PCR results at any of the 3 genetic loci (*bg*, *tpi* and *gdh*), 126 (41.2%) of the 306 faecal specimens tested positive for *G*. *duodenalis*, which was similar to the occurrence in Hubei (37.8%, 28/74) [16], but much higher than the majority of provinces in China. These provinces included Jilin (4.4%, 5/114) [14], Liaoning (3.1%, 3/98) [14], Heilongjiang (12.5%, 3/24) [14], Shaanxi (17.54%, 30/171) [20], Xinjiang (16.6%, 46/277) [15] and Guangdong (1.1%, 5/533) [17]. Compared with studies in other countries, the overall infection rate in the present study was higher than in Maryland, USA (32.1%, 125/390) [11]; Malaysia (8.3%, 10/120) [8]; India (12.5%, 9/72) [10] and New Zealand (2%, 2/100) [9], but lower than that in another study in the USA (52%, 237/456) [12]. The reasons for these differences in infection rates is still unclear, however, they may be related to geo-ecological conditions, management factors and health status [1, 15, 21, 22]. Nested PCR amplification is a sensitive method and widely used in the detection of *G*. *duodenalis* [3, 6]. In this study, nested PCR amplification was used directly instead of using microscopic examination which led to low detection and also cannot genotype *G*. *duodenalis*. Numerous studies have reported that the prevalence of *G*. *duodenalis* is inversely associated with animal age [1, 8, 11]. However, we measured a significantly higher occurrence of *G*. *duodenalis* in post-weaned calves (41.2%) compared with previous measurements of pre-weaned calves (26/278, 9.4%) [18] (*P* < 0.01, *X*^2^ = 76.623, *df* = 1); which was similar to some reports from China [15, 16] and the USA [12, 13]. Moreover, we analysed the infection rates between intensive feeding and free-ranging farms and found consistent results with our previous study [18], i.e., no significant differences between the two breeding systems (*P* = 0.179, *X*^2^ = 1.809, *df* = 1). Similarly, a study conducted in Malaysia also showed no significant differences in the occurrence of *G*. *duodenalis* infection between intensive and semi-intensive farms [8].

**Table 1:** Occurrence and assemblages of *Giardia duodenalis* in post-weaned dairy calves in Sichuan province.

Region	Number of tested	Number of positive specimens/ *G*. *duodenalis* assemblage (n)			*G*. *duodenalis* infection rate (Number of positive specimens combining three gene loci)/ 95% confidence intervals (CI)
		*bg*	*tpi*	*gdh*	
Anyue^b^	30	19/E(19)	17/ A(1), E(16)	17/E(17)	63.3% (19)/ 45.0~81.6
Chengdu^a^	32	15 /A(2), E(13)	14/ A(2), E(12)	14/ A(2), E(12)	46.9% (15)/ 28.6~65.2
Deyang^b^	20	8/E(8)	8/E(8)	8/E(8)	45.0% (9)/ 21.1~68.9
Meishan^b^	31	15/E(15)	9/E(9)	12/E(12)	51.6% (16)/ 33.0~70.2
Mianyang^a^	36	20/A(3), E(17)	16/A(1), E(15)	18/A(1), E(17)	58.3% (21)/ 41.4~75.3
Nanchong^a^	30	13/E(13)	11/E(11)	11/E(11)	43.3% (13)/ 24.5~62.2
Qingbaijiang^b^	31	13/E(13)	11/A(1), E(10)	12/E(12)	41.9% (13)/ 23.5~60.3
Qionglai^a^	41	14/A(1), E(13)	12/A(1), E(11)	13/A(1), E(12)	34.1% (14)/ 19.0~49.3
Xichang^b^	30	0	0	2/E(2)	6.7% (2)/ -2.8~16.1
Ya’an^b^	25	4/E(4)	3/E(3)	4/E(4)	16.0% (4)/ 0.6~31.4
Total	306	121/A(6), E(115)	101/A(6), E(95)	111/A(4), E(107)	41.2% (126)/ 35.6~46.7

^a^: intensive farming

^b^: free-ranging

Of the 126 *G*. *duodenalis*-positive specimens, 121 were positive in *bg* gene, 101 in *tpi* gene, and 111 in *gdh* gene. *Giardia duodenalis* assemblage E (n = 115, 91.3%), assemblage A (n = 3, 2.4%), and mixed assemblage (A and E) (n = 8, 6.3%) were identified among the 126 genotyped specimens (Table 1), which is consistent with other studies conducted in China [14], the USA [11] and India [10]. All of the assemblage A isolates identified in the present study belonged to subtype A1, which has mostly been detected in animals [1, 5]. However, there have been a few reports of human infections of subtype A1, e.g., in China [23], Portugal [24], Mexico [25] and Brazil [26], which suggests that dairy calves may potentially play a role in the zoonotic transmission of *G*. *duodenalis* infection from cattle to humans. Sequence analyses revealed 12, 9 and 15 subtypes identified at the *bg*, *tpi* and *gdh* loci, respectively. Of the *bg* subtypes, eight had previously been identified. The remaining four sequences represented subtypes E17-E20 (MK642904-MK642907) that were previously unpublished. Of the *tpi* subtypes, eight subtypes identified before and the remaining one sequence subtype E25 (MK642913) was previously unpublished. Of the *gdh* subtypes, ten were known and five were previously unpublished: E21–E25 (MK642908-MK642912). Of these subtypes, the most common were E9 (n = 20, *bg* subtype), E3 (n = 64, *tpi* subtype) and E10 (n = 48, *gdh* subtype). The high genetic diversity of assemblage E in this study was consistent with previous studies [18, 27, 28], which may be related to intra-assemblage genetic recombination [19, 29].

Furthermore, MLG analysis of the *bg*, *tpi* and *gdh* genes was used to systematically characterize intra-assemblage genetic diversity and to determine the genotype of *G*. *duodenalis*. A total of 94 specimens were successfully sequenced at the *bg*, *tpi* and *gdh* loci, which formed 34 assemblage E MLGs (Table 2), one assemblage A MLG and seven mixed assemblage (A and E) MLGs. The most common MLGs were the MLG E48 (n = 11) and MLG E80 (n = 11), followed by MLG E94 (n = 9) and MLG E3 (n = 6). Among them, the most widely distributed MLG was MLG E3, which was detected in four regions (Chengdu, Deyang, Meishan and Ya’an). To reveal the genetic relationship between MLGs, we constructed clonal pedigree maps of the 34 assemblage E MLGs in the present study and the 19 assemblage E MLGs in our previous study [18] using eBURST software. Two clonal complexes and five singletons were observed (S1 Fig). MLG E3 is the primary founder of clonal complex 1, which is consistent with the results found in Shanghai [28]. Clonal complex 2 was formed by three assemblages E: MLG E94-E96. MLG E69, MLG E78, MLG E83, MLG E87 and MLG E98 were singletons. To better understand the diversity between assemblage E MLGs in Sichuan and in the other provinces of China (Henan [30], Gansu [31], Ningxia [31], Shaanxi [20], Xinjiang [15], Shanghai [28] and Guangdong [17, 27]), a phylogenetic evolutionary tree was constructed. The phylogenetic tree (S2 Fig) showed that MLG E3 has also been found in Gansu [31], Xinjiang [15], Shanghai [28] and Guangdong [27], which indicates the wide distribution of this MLG subtype in China. However, the predominant subtype needs further elucidation. As seen from the phylogenetic tree, the 218 assemblage E MLGs identified in China were highly diverse and formed multiple evolutionary branches. The 53 assemblage E MLGs from Sichuan province showed a scattered distribution in the phylogenetic tree, which indicated that geographical segregation was not strict in our study.

**Table 2:** Multilocus sequence genotypes of *Giardia duodenalis* assemblage E in post-weaned dairy calves in Sichuan province.

Isolate	Geographic source	Subtype			MLGs
		*bg*	*tpi*	*gdh*	
AY01,15,22,23,26	Anyue	#E17/MK642904	E3/KT922259	E10/KT698971	#MLG E76
AY02		E8/KY769093	E3	E2/KY769098	#MLG E77
AY03,04,21,24,25,28,30		E8	E3	E10	MLG E48
AY08,29		#E17/MK642904	E7/KT369768	E1/KY769096	#MLG E78
AY27		E9/KY769091	E7	E10	#MLG E79
CD04,08,13,20,21,25	Chengdu	E2/KT922248	E3	E10	#MLG E80
CD05,29		E1/KT922247	E3	E10	#MLG E81
CD06		E9	E6/KY432850	E10	#MLG E82
CD09		E2	E6	#E21/MK642908	#MLG E83
CD27		E9	E3	E10	MLG E3
DY02	Deyang	E9	E3	E10	MLG E3
DY06		E9	E5/MH893667	E1	#MLG E84
DY09		E9	E3	E14/KY769097	MLG E36
DY11		#E18/MK642905	E3	E9/KY710742	#MLG E85
DY14		E3/KT369773	E3	E8/KT369785	#MLG E86
DY17		E3	E5	E10	#MLG E87
MS11	Meishan	E9	E3	E3/KT369780	#MLG E88
MS13		E11/KY769089	E3	#E22/MK642909	#MLG E89
MS16,19,27		E9	E3	E10	MLG E3
MS25		E2	E3	#E23/MK642910	#MLG E90
MY03	Mianyang	E2	E22/KY710748	E10	#MLG E91
MY10,15,23,25,35		E2	E3	E10	#MLG E80
MY21,29,32,33		E8	E3	E10	MLG E48
MY24		E8	E3	#E22/MK642909	#MLG E92
MY34		E8	E3	E12/KY432840	#MLG E93
NC02,12,13,14,17,18,19,24,26	Nanchong	E2	E4/KY769102	E1	#MLG E94
NC15		E2	#E25/MK642913	#E24/MK642911	#MLG E95
NC16		E2	#E25/MK642913	E1	#MLG E96
QBJ06,09,17,28,31	Qingbaijiang	E11	E3	E10	MLG E21
QBJ12		E13/MF671880	E3	E10	#MLG E97
QBJ18		E11	E22	#E25/MK642912	#MLG E98
QBJ21,25,26		E11	E3	E8	#MLG E99
QL12,14,23,34,35	Qionglai	E9	E15/KY432848	E1	MLG E4
QL17		E11	E15	E12	#MLG E100
QL18,36,39,40		E13	E15	E1	#MLG E101
QL41		E11	E15	E1	MLG E20
YA14	Ya’an	E9	E3	E9	#MLG E102
YA18		E9	E3	E2	#MLG E103
YA22		E9	E3	E10	MLG E3

# Novel subtypes and novel MLGs

## Conclusion

The present study demonstrated a high occurrence of *G*. *duodenalis* in post-weaned calves in Sichuan province, China. Both assemblage E and zoonotic assemblage A were detected. MLG analysis revealed a high genetic diversity in assemblage E. MLG E3 was shown to be not only the primary MLG subtype in Sichuan province but also the most widely distributed MLG subtype throughout China.

## Supporting information

S1 Fig eBURST networks for *G*. *duodenalis* assemblage E isolated from Sichuan province. Each MLG is represented by a dot. MLG-E3 is the primary founder, and the subgroup founders are MLG-E21, MLG-E48, MLG-E61, MLG-E65, MLG-E74, MLG-E80, MLG-E81 and MLG-E97. The variants are connected by lines.
(TIF)

S2 Fig Phylogenetic relationships between *Giardia duodenalis* assemblage E MLGs. The phylogenetic tree was constructed using a concatenated dataset of *bg*, *tpi* and *gdh* gene sequences, bootstrap values greater than 50% from 1000 replicates are shown. Isolates from the present study are indicated by black triangles, and isolates from our previous study [18] are indicated by black circles.
(TIF)
