# Supplementary figures and images for: Occurrence and multilocus genotyping of Giardia duodenalis from post-weaned dairy calves in Sichuan province, China
Source: PLoS One. 2019 Nov 4;14(11):e0224627. doi: 10.1371/journal.pone.0224627 (PMC6827885; doi:10.1371/journal.pone.0224627)

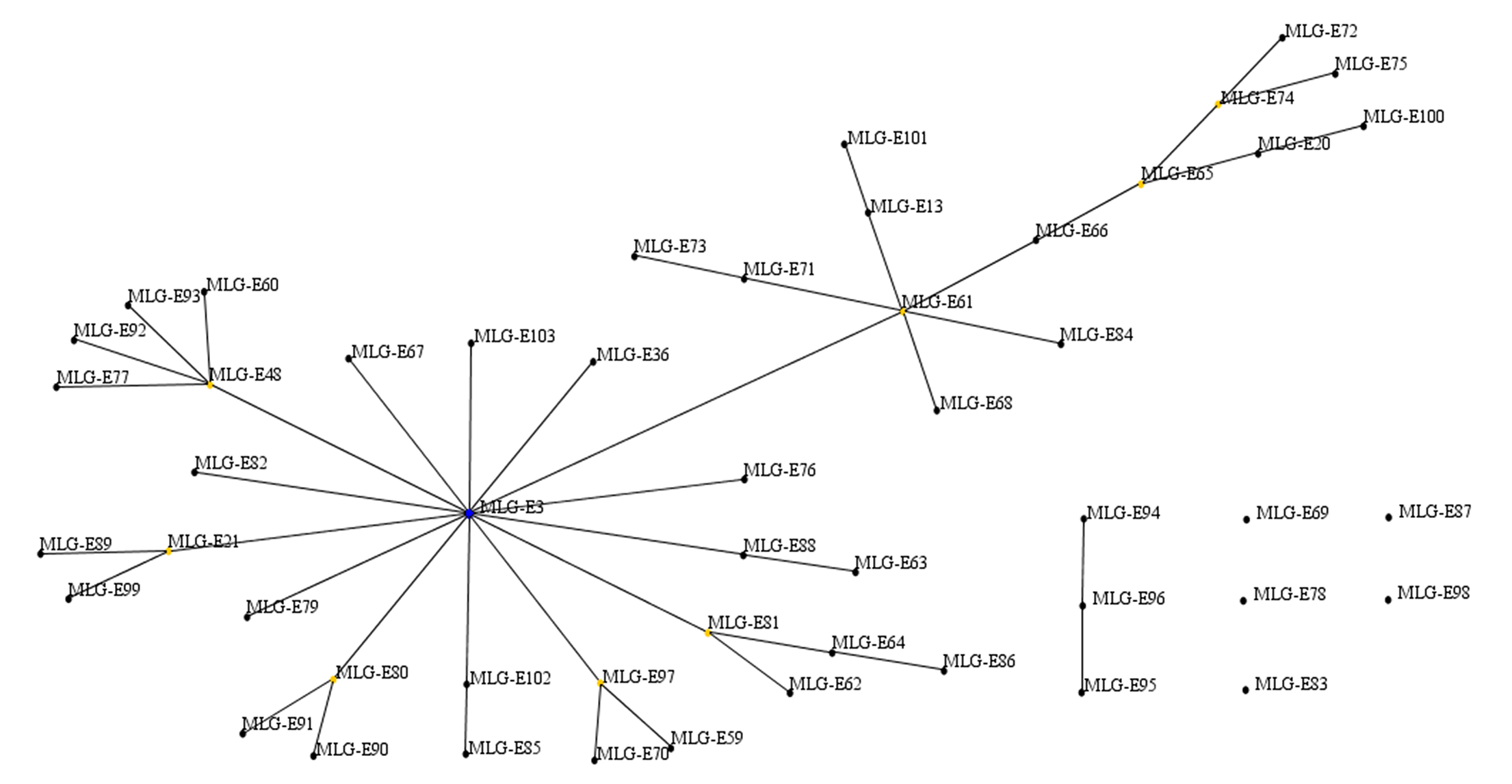

Supplement: S1 Fig — Each MLG is represented by a dot. MLG-E3 is the primary founder, and the subgroup founders are MLG-E21, MLG-E48, MLG-E61, MLG-E65, MLG-E74, MLG-E80, MLG-E81 and MLG-E97. The variants are connected by lines. (TIF) [file pone.0224627.s001.tif]

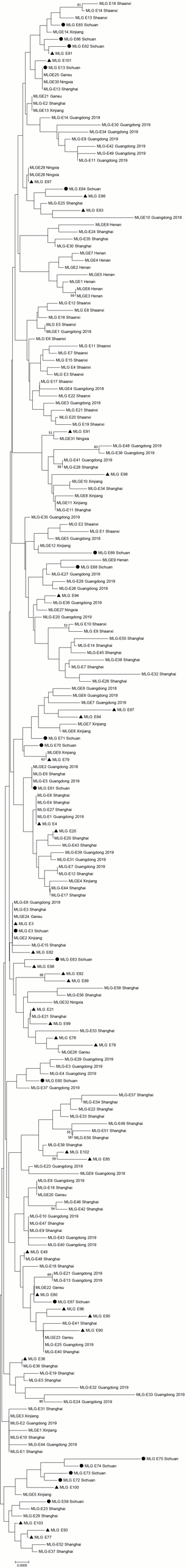

Supplement: S2 Fig — The phylogenetic tree was constructed using a concatenated dataset of bg, tpi and gdh gene sequences, bootstrap values greater than 50% from 1000 replicates are shown. Isolates from the present study are indicated by black triangles, and isolates from our previous study [18] are indicated by black circles. (TIF) [file pone.0224627.s002.tif]
